# Supplementary material for: Clocked dynamics in artificial spin ice
Source: Nat Commun. 2024 Feb 1;15:964. doi: 10.1038/s41467-024-45319-7 (PMC10834408; doi:10.1038/s41467-024-45319-7)
Supplement: Supplementary file 1 — Supplementary Information [file 41467_2024_45319_MOESM1_ESM.pdf]

Supplementary information: Clocked dynamics in artificial spin ice

Johannes H. Jensen[1*†], Anders Strømberg[2*†], Ida Breivik[2], Arthur Penty[1], Miguel Angel Niño[3], Muhammad Waqas Khaliq[3], Michael Foerster[3], Gunnar Tufte[1], Erik Folven[2]

[1*]Department of Computer Science, Norwegian University of Science and Technology, Trondheim, Norway.
[2*]Department of Electronic Systems, Norwegian University of Science and Technology.
[3]ALBA Synchrotron Light Facility, Carrer de la Llum 2 – 26, Cerdanyola del Vallés, 08290, Barcelona, Spain.

*Corresponding author(s). E-mail(s): johannes.jensen@ntnu.no; anders.stromberg@ntnu.no;
†These authors contributed equally to this work.

1 Supplementary Figures

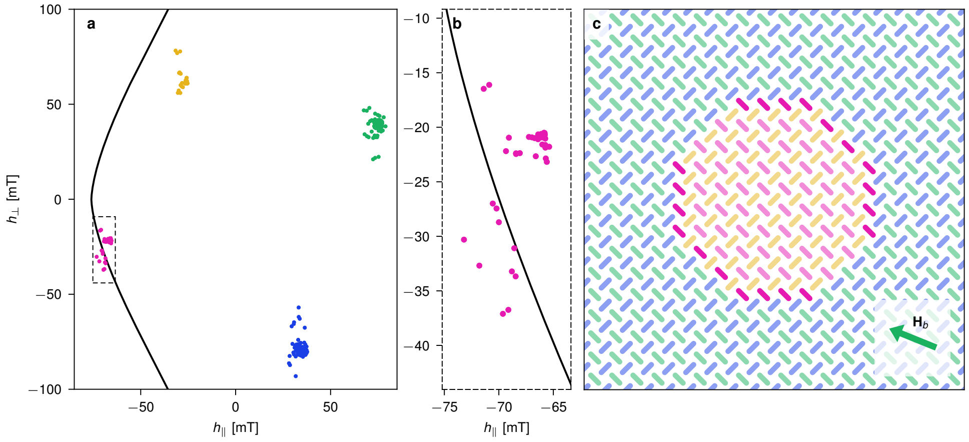

Fig. 1 Astroid clusters showing relative locations of all the magnets within their respective switching astroids. The plots show **a-b**, astroid clusters during reversal, when the pinwheel system shown in **c** is subject to the negative clock field $\mathbf{H}_b$. Each dot represents the total field $\mathbf{h}_i = \mathbf{H}_b + \mathbf{h}_{\text{dip}}^{(i)}$ experienced by a magnet i, projected onto its parallel ($h_{||}$) and perpendicular ($h_{\perp}$) axis. Note that the positive direction of the parallel component is with respect to the magnetization direction of each nanomagnet. **b**, astroid clusters during reversal have a different structure compared to growth. Switchable magnets outside the astroid are highlighted in **c**. During reversal, the switchable magnets are along both the horizontal, vertical and $-45°$ domain walls. Switchable magnets along the horizontal domain wall is attributed to the curvature of the inner domain.

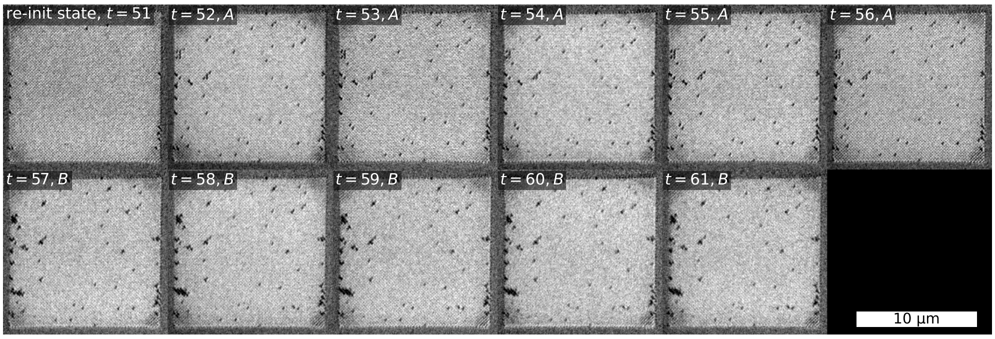

Fig. 2 XMCD-PEEM images of the control experiment. The system is reinitialized at $t = 51$ (following from Fig. 7 in the main text), and t is incremented by 1 for each clock step, with clock pulses indicated by the labels. The first A clock pulse promotes dark (rightwards) magnets, equivalent to half a clock cycle, while subsequent applications of A incurs no further change. When the clock pulse is changed to B, dark (rightwards) magnets are again promoted, equivalent to the second half of an AB clock cycle. Furthermore, additional B clock pulses incurs no change in the state.

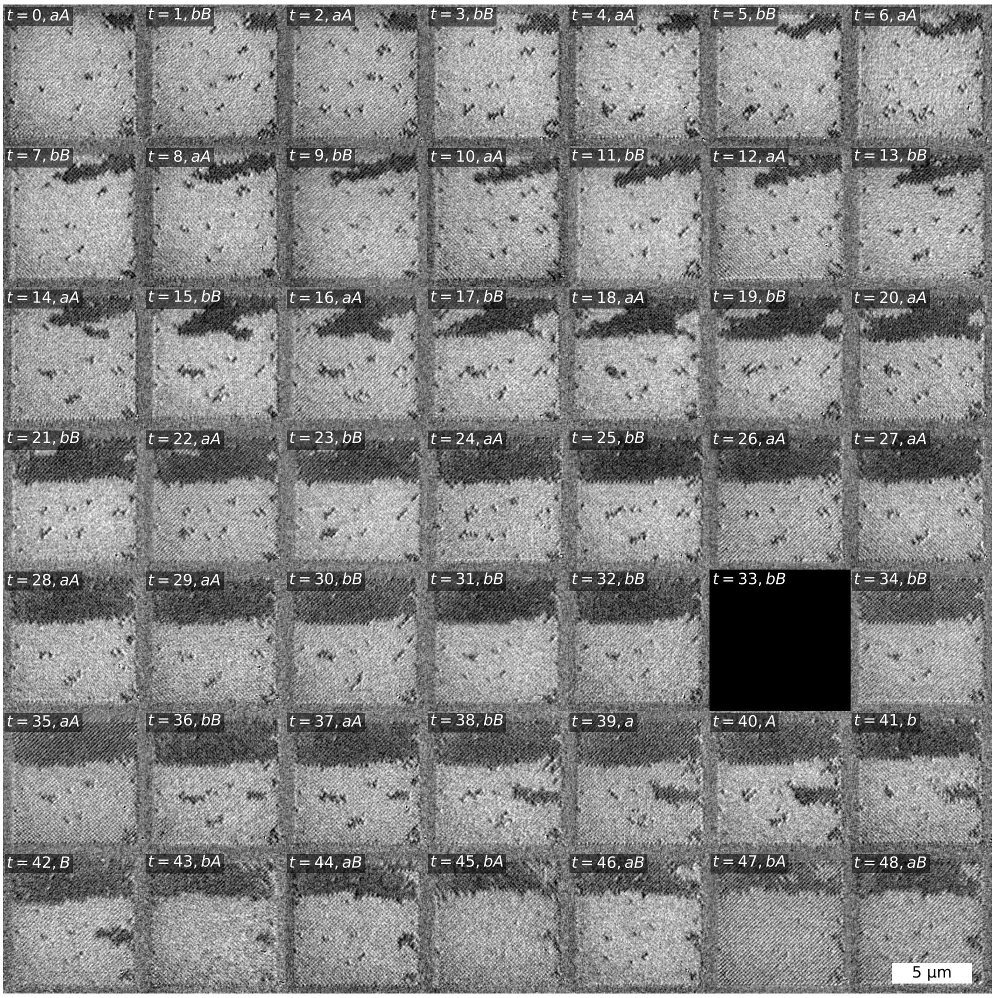

Fig. 3 XMCD-PEEM images of all steps from the bipolar clock protocol series. The time starts at $t = 0$, and is incremented by 1 for each image, with clock pulses indicated by the labels. The black (rightwards) domains grow and change shape as the $aAbB$ protocol is applied. There are two control series where aA and bB are applied, where no change occurs. Note that there is missing data for $t = 33$, but the ensemble was still subjected to the clock pulses. At $t = 39$ we image after each single clock pulse. From $t = 42$ the reverse protocol $BbAa$ is applied, and the black (rightwards) domains shrink. Note that during the reversal protocol we still image after a final A or B pulse, in order to keep a constant image shift.

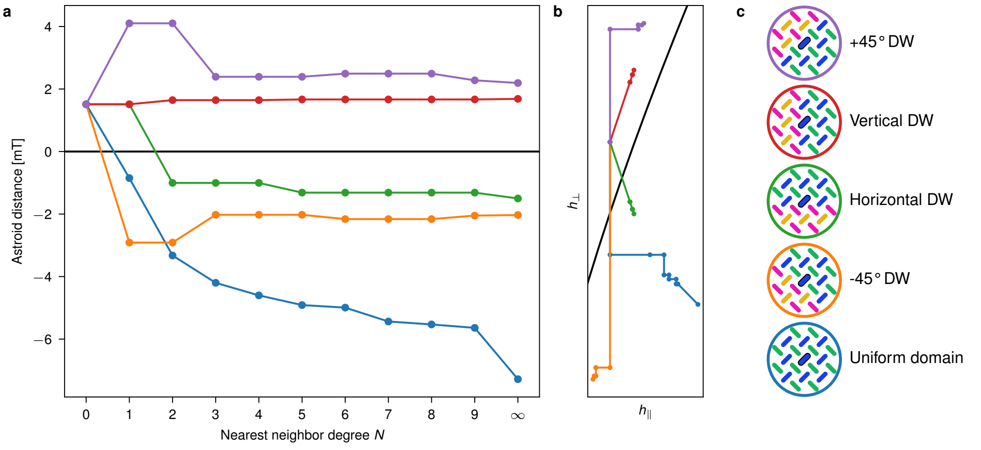

Fig. 4 Minimum distance to the astroid edge as the neighborhood is increased, for the highlighted blue magnet in the center of each scenario in **c**. Each scenario is color-coded according to the circles in **c**. In all cases, the clock field $\mathbf{H}_A$ is applied, as defined in Fig. 1 in the main text. **a**, distance to the astroid for the highlighted magnet, as the neighborhood is increased when calculating the dipolar fields. For each nearest neighbor degree N, the dipolar fields include all magnets within a radius of the Nth nearest neighbor. **b**, trace of the position within the astroid as the nearest neighbor degree N is increased. Each dot represents the total field projected onto the parallel ($h_{||}$) and perpendicular ($h_\perp$) axis of the blue center magnet. The positive direction of the parallel component is with respect to the magnetization direction of the nanomagnet. Note that the scenarios all start at the same point (no neighbors), then diverge.

2 Supplementary Discussion

2.1 Neighborhood interactions

Here we analyse what type of neighbor interactions causes switching to occur selectively along the vertical and $+45°$ domain walls. We consider five different prototype cases shown in Fig. 4c: a uniform blue/green (leftwards) domain, and two domains separated by horizontal, vertical, and $\pm45°$ domain walls (DWs). Within each prototype case, the subject of study is the highlighted blue magnet in the center. The circled insets in the figure show only a limited neighborhood in the center of a larger 50×50 system which is initialized according to each prototype case.

Fig. 4a plots the distance to the astroid for the center magnet, as the number of neighbors are increased when calculating the dipolar fields. For each nearest neighbor degree N, the dipolar fields include all magnets within a radius of the Nth nearest neighbor (NN). After adding the total dipolar field to the external clock field H_A, the shortest distance to the astroid is calculated. We define astroid distance as positive outside the astroid and negative inside.

Astroid distance is plotted for each of the five prototype cases in Fig. 4c. With zero neighbors, and hence no dipolar fields, all five cases start at the same point outside the astroid. As the first NNs are included, the cases split into four: the uniform domain and the $-45°$ domain wall enter the astroid. In other words, the dipolar fields from the first NNs stabilize and prevent switching in these two cases. Including also the second

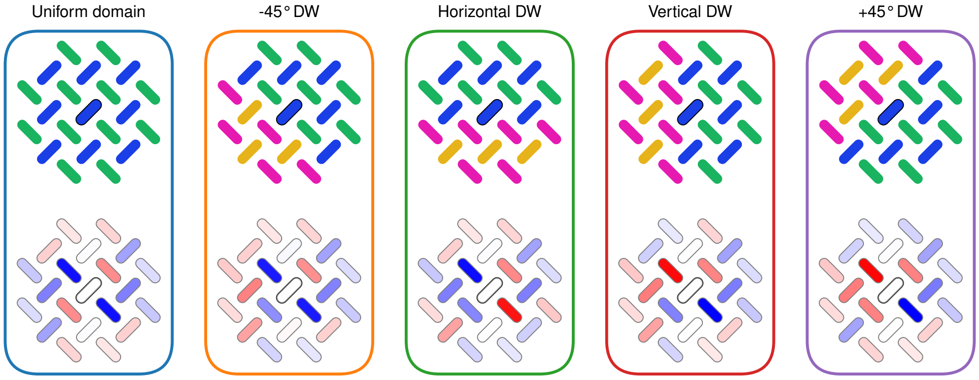

Fig. 5 Neighborhood influence with respect to center magnet, for nearest neighbor degree 1-4. Each scenario depicts the magnetization state (top) and the corresponding influence of the neighbors (bottom). In all cases, the clock field $\mathbf{H}_A$ is applied, as defined in Fig. 1 in the main text. Stronger red signifies that the magnet is biasing the center magnet *towards* switching, and stronger blue signifies that the magnet is biasing the center magnet *away* from switching.

NNs causes the horizontal domain wall to enter the astroid. Horizontal domain walls are hence stabilized by 2nd NN interactions. For the horizontal and -45° domain walls, astroid distance does not change significantly as the neighborhood is increased further. For the uniform domain, however, astroid distance increases further as the number of NNs are increased, with significant stabilizing interactions also beyond 9NNs.

Next, we consider the two cases where switching *does* occur, namely the vertical and $+45^\circ$ domain walls. Somewhat curious, the astroid distance for the vertical domain wall appears to stay nearly constant across all NNs. The $+45^\circ$ domain walls travel further outside the astroid due to 1st NN interactions, then the 3rd NN interactions bring it closer to the astroid again, after which it remains at a near-constant distance.

Fig. 4b shows a trace of the location within the astroid as the NNs are increased. For the vertical domain wall (red line), there is indeed movement due to dipolar interactions, but the movement is exclusively *parallel* to the astroid edge. Hence, the astroid distance in this case remains constant. For the $+45^\circ$ domain wall (purple line), the movement is purely in the perpendicular ($h_\perp$) direction for the 1st NN interactions, then purely parallel ($h_\parallel$) from the 3rd NN fields.

An even more detailed picture is provided in Fig. 5, where each neighbor magnet is colored according to the contribution of its dipolar field. Specifically, a magnet is colored red (blue) if its dipolar field pushes the center magnet further out of (into) the astroid. The shade of red (blue) represents how much the dipolar field contributes to promote (prevent) switching of the center magnet. A magnet is colored white if its dipolar field has no contribution on the resulting astroid distance.

As can be seen in Fig. 5, the neighborhood in the uniform domain is dominated by magnets that prevent switching (colored blue), with the highest contribution from the first NNs along the hard axis of the center magnet. The same subset of the NNs are also the primary stabilizing force of the -45° DW. For the horizontal DW case,

the dipolar fields from the first NNs cancel out, and it is the second NNs that prevent switching.

For the vertial DW, there is an apparent symmetry between neighbors that prevent and promote switching. As a result the vertical DW is not stabilized and hence easily switched. We saw earlier how this is because the dipolar fields are directed parallel to the astroid edge. The +45° DW is the least stable, where 3/4 of the first NNs promote switching (colored red).

2.2 Growth and reversal in bipolar clocking

During bipolar clocking, domain growth and reversal in a single clock cycle can be observed for several domain wall configurations. Fig. 6 shows the time evolution of different types of domain walls, subject to $aAbB$ clocking. A straightforward example of simultaneous growth and reversal can be seen in Fig. 6d, which shows a +45° domain wall. Notice that the first clock pulse a moves the domain wall one step towards the left, and hence a reversal of the orange/pink domain. However, the subsequent A pulse immediately undoes this change *and* moves the domain wall another step towards the right, advancing the domain wall a total of two layers of the sublattice L_a (orange magnets). Next, the b pulse has no effect, since the pink magnets along the domain wall are stabilized by the dipolar fields from their neighbors. Finally, the B pulse moves the domain another step towards the right, flipping the next layer of magnets from sublattice L_b (from green to pink). As can be seen, the result is an apparent growth of the orange/pink domain by a single layer along the domain wall. The other domain wall cases in Fig. 6 also show simultaneous growth and reversal, but are not discussed in further detail.

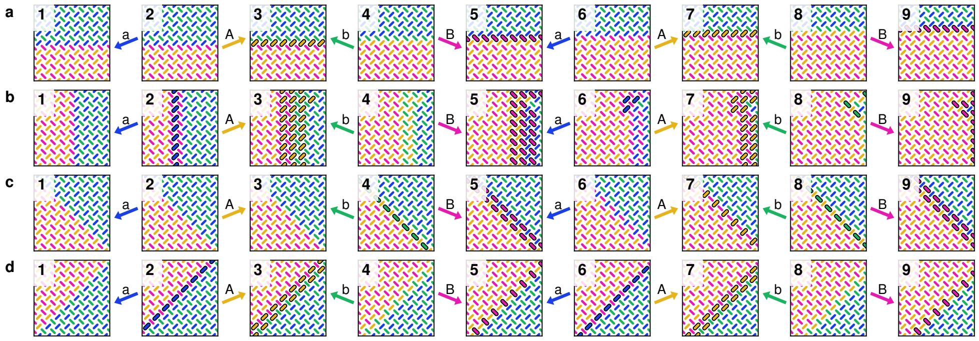

Fig. 6 Bipolar $aAbB$ clocking of four types of domain walls in pinwheel ASI: **a**, horizontal DW, **b**, vertical DW, **c**, −45° DW and **d**, +45° DW. Each domain wall is initialized to fill the whole 50×50 system from edge to edge. Each snapshot shows a zoomed-in view of the system, at different points during a clock protocol. (1) shows the initial state. (2-12) show the state during $aAbB$ clocking. Magnets that change state between snapshots are highlighted by a solid black outline.

There is an apparent competition between growth and reversal. For the +45° domain wall discussed earlier, the competition seems to favor growth. However, the

situation strongly depends on the particular shape of the domain. Fig. 10 in the main text shows the time evolution of a hexagonal domain subject to $aAbB$ clocking. As can be seen, the domain both grows horizontally and reverses vertically, and hence gradually changes shape over time. Since vertical domain reversal depends on the curvature of the domain, the process will stop when the domain grows too wide. The domain will continue to grow horizontally, as horizontal domain growth is not dependent on curvature. As a result, domain growth seems to out-compete reversal in this case. The end result is an apparent tendency towards horizontally elongated domains.
