# Supplementary material for: Clocked dynamics in artificial spin ice
Source: Nat Commun. 2024 Feb 1;15:964. doi: 10.1038/s41467-024-45319-7 (PMC10834408; doi:10.1038/s41467-024-45319-7)
Supplement: Supplementary file 2 — Description of Additional Supplementary Files [file 41467_2024_45319_MOESM2_ESM.pdf]

Title: Supplementary Movie 1:
Description: Video showing all XMCD-PEEM images from the unipolar clock protocol series. Each frame is labeled by the clock pulse(s) preceding it. The series consists of growth, reversal, reinitialization, control experiment and a second phase of growth.

Title: Supplementary Movie 2:
Description: Video showing flatspin simulation of the unipolar clock protocol series. Each frame depicts the clock pulse preceding it. The series consists of growth, reversal, re-initialization and control experiment.

Title: Supplementary Movie 3:
Description: Video showing all XMCD-PEEM images from the bipolar clock protocol series. Each frame is labeled by the clock pulse(s) preceding it. The series consists of growth, control experiment and reversal.

Title: Supplementary Movie 4:
Description: Video showing flatspin simulation of the bipolar clock protocol series. Each frame depicts the clock pulse preceding it. The series consists of growth, control experiment and reversal.
